# Mitophagy programs: mechanisms and physiological implications of mitochondrial targeting by autophagy

**DOI:** 10.1007/s00018-015-2087-8

**Published:** 2015-11-26

**Authors:** Anne Hamacher-Brady, Nathan Ryan Brady

**Affiliations:** 1 Lysosomal Systems Biology, German Cancer Research Center (DKFZ), Heidelberg, Germany; 2 Systems Biology of Cell Death Mechanisms, German Cancer Research Center (DKFZ), Heidelberg, Germany; 3 Department of Surgery, Heidelberg University Hospital, Heidelberg, Germany; 4 Bioquant, University of Heidelberg, INF 267, BQ0045, 69120 Heidelberg, Germany

**Keywords:** Bnip3, FUNDC1, LC3-interacting region (LIR), Macroautophagy, Mitophagy, Nix, Parkin E3 ligase, Ubiquitin

## Abstract

Mitochondria are an essential source of ATP for cellular function, but when damaged, mitochondria generate a plethora of stress signals, which lead to cellular dysfunction and eventually programmed cell death. Thus, a major component of maintaining cellular homeostasis is the recognition and removal of dysfunctional mitochondria through autophagy-mediated degradation, i.e., mitophagy. Mitophagy further constitutes a developmental program, and undergoes a high degree of crosstalk with apoptosis. Reduced mitochondrial quality control is linked to disease pathogenesis, suggesting the importance of process elucidation as a clinical target. Recent work has revealed multiple mitophagy programs that operate independently or undergo crosstalk, and require modulated autophagy receptor activities at outer membranes of mitochondria. Here, we review these mitophagy programs, focusing on pathway mechanisms which recognize and target mitochondria for sequestration by autophagosomes, as well as mechanisms controlling pathway activities. Furthermore, we provide an introduction to the currently available methods for detecting mitophagy.

## Introduction

**Fig. 1:**
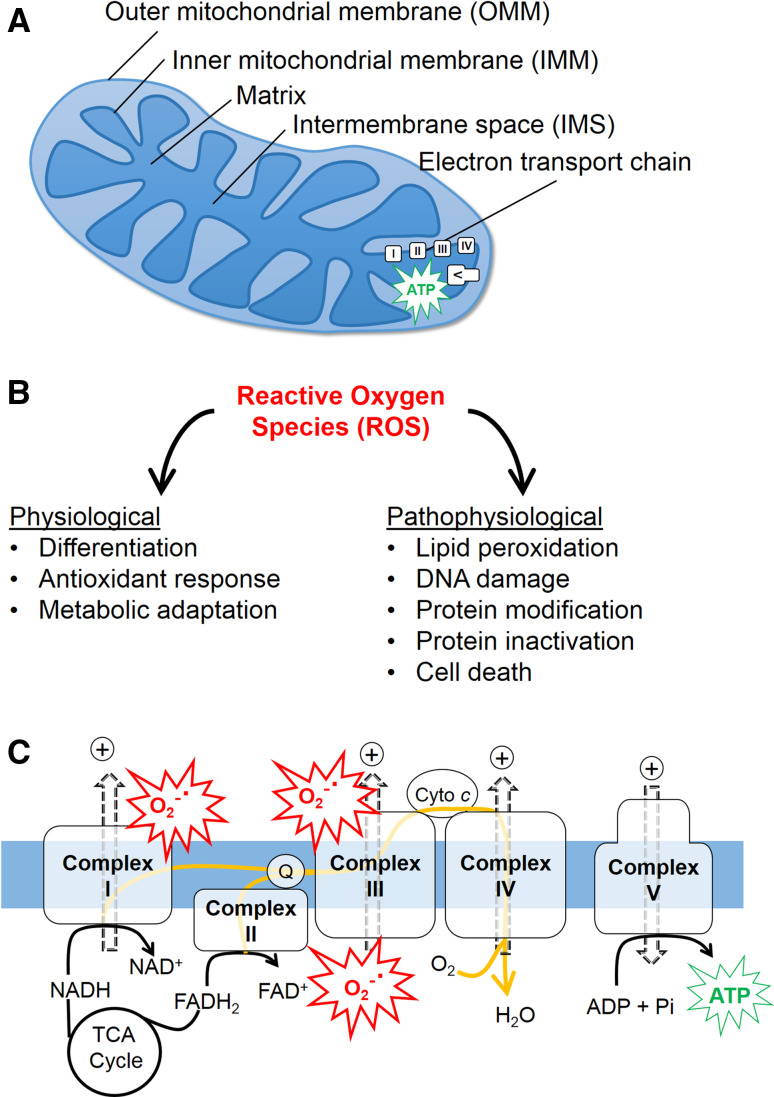
Mitochondrial function and dysfunction. **a** A mitochondrion is enclosed by two membranes, the outer mitochondrial membrane (OMM) and inner mitochondrial membrane (IMM). The mitochondrial compartment between the OMM and IMM is referred to as the intermembrane space (IMS). The respiratory chain (electron transport chain, ETC), which is composed of complexes I–V and localized in the IMM, drives ATP synthesis in the mitochondrial matrix. **b** ROS are implicated in both physiological and pathophysiological signaling. **c** Electrons from the tricarboxylic acid (TCA) cycle substrates are transferred through the respiratory chain complexes (along the *yellow arrows*), driving the extrusion of protons (+) from the matrix, thereby generating the proton motive force. Proton flow through the ATP synthase (complex V) drives ATP production. Oxygen (O_2_) serves as the terminal electron acceptor at complex IV, forming H_2_O. At complexes I and/or III electron leak can produce the reactive oxygen species (ROS) superoxide anion (O_2_^−^˙)

Mitochondria are organelles surrounded by a double membrane, comprised of the outer mitochondrial membrane (OMM) and the inner mitochondrial membrane (IMM) (Fig. 1a). Mitochondria are abundant in most cell types, and occupy approximately 10–40 % of cellular volume [1]. For example, the mitochondrial population accounts for approximately 30 % of the cellular volume of HeLa cells, and for 22–37 % of the cardiac cell volume [2]. Furthermore, depending on cell types, mitochondrial morphologies [3] and numbers vary considerably. For example, cardiac myocytes contain several thousand morphologically similar mitochondria [4], while neurons carry several hundred dynamic and morphologically heterogeneous mitochondria [5]. The cellular functions of mitochondria are multifaceted and encompass the production of the bioenergetic carrier adenosine triphosphate (ATP), participation in reactive oxygen species (ROS) (Fig. 1b) [6] and calcium [7] signaling, metabolite synthesis, programmed cell death [8], and tumorigenesis [9, 10].

Mitochondrial bioenergetic function involves the oxidation of acetyl-CoA in the tricarboxylic acid (TCA) cycle to generate NADH and FADH_2_, which transfer electrons to the electron transport chain to produce an electrochemical gradient across the IMM that is used to produce ATP [11] (Fig. 1c). Ultimately, electrons are transferred to molecular oxygen (O_2_), reducing it to H_2_O. The coupling of these processes is termed mitochondrial oxidative phosphorylation, aka cellular respiration. Due to leakage of electrons at complex I or complex III of the electron transport chain, O_2_ can be incompletely reduced and generate the superoxide anion, the precursor to most ROS [12]. Low levels of ROS play physiological roles [6], while high and/or prolonged elevations of ROS can oxidize proteins, lipids, and nucleic acids, leading to cellular dysfunction and programmed cell death [13].

Damaged mitochondria can signal programmed cell death [8], inflammation and aging [14]. Enhanced levels of damaged mitochondria aggravate many diseases, and participate in disease pathogenesis [15–17]. To maintain homeostasis of the mitochondrial population, cells rely on autophagy, a quality control process by which components of the cytoplasm are sequestered and delivered to lysosomes for degradation [18]. Dysfunctional mitochondria can be recognized and targeted for degradation by a specific mode of autophagy, termed mitophagy. Experimentally this has been evidenced in cell culture and in vivo models, whereby genetic knockout of autophagy proteins results in increased mitochondrial mass, and increased numbers of dysfunctional mitochondria and ROS levels [19, 20]. While targeting of mitochondria to lysosomes was detected over 50 years ago [21], recent years have yielded rapid progress in elucidating mechanisms which underlie distinct mitophagy programs. In this review, we focus on the mechanisms which orchestrate the targeting of the autophagic machinery to the OMM, discuss physiological roles of mitophagy, and present an overview of methods used to evidence mitophagic activity.

## Mitochondrial dynamics prime mitochondria for mitophagy

**Fig. 2:**
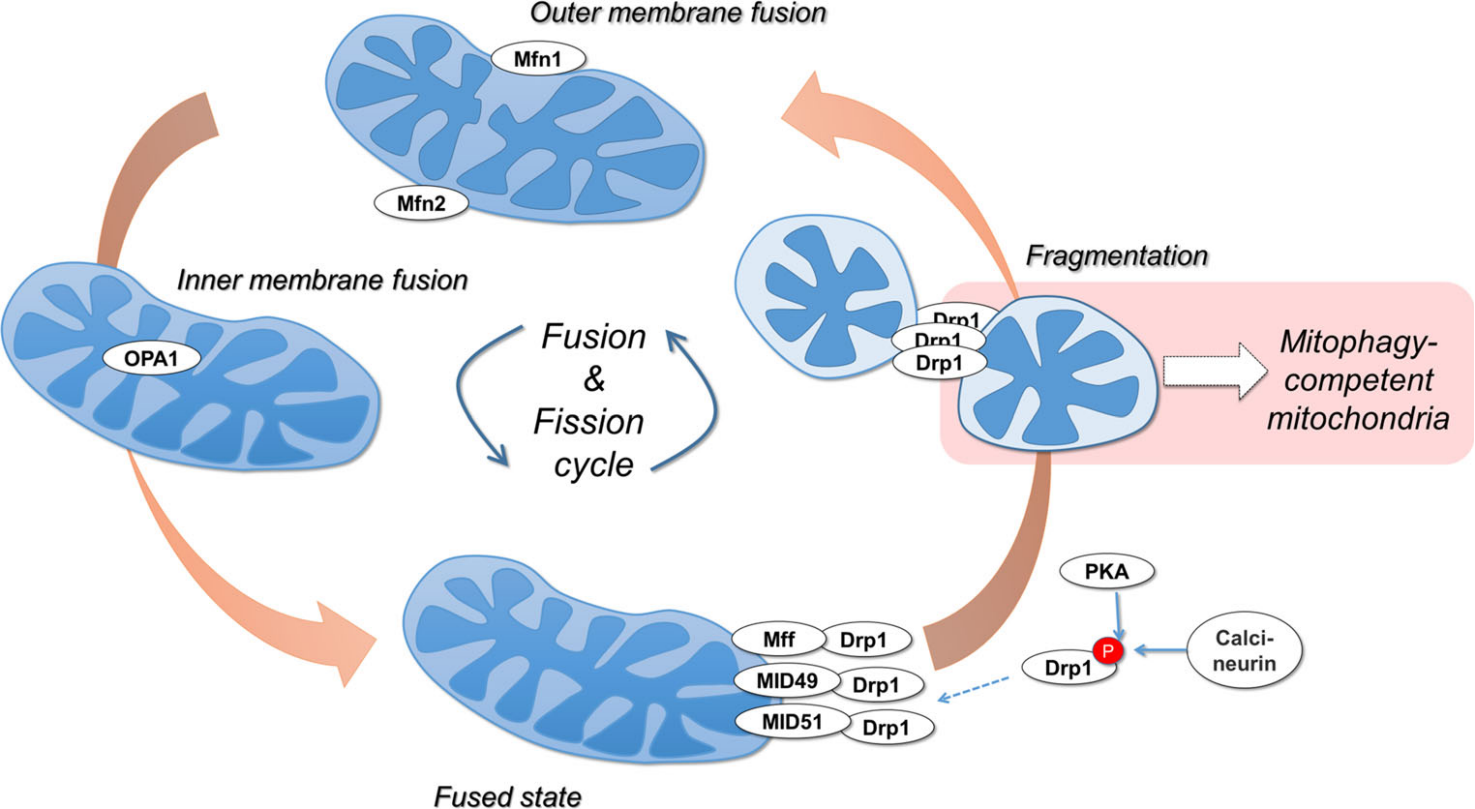
Mitochondrial dynamics are linked to mitophagy. The GTPases Mitofusin 1 (Mfn1) and Mitofusin 2 (Mfn2) mediate OMM fusion, and the GTPase OPA1 (Optic atrophy 1) mediates IMM fusion. Mitochondrial fragmentation requires the translocation of the GTPase Drp1. Normally cytosolic, Drp1 is recruited to mitochondria via OMM-bound receptor proteins Fis1, Mff, MID49, and MID51. Protein kinase A (PKA)-mediated phosphorylation of Drp1 at serine 656 inhibits its activity, resulting in hyperfused mitochondrial networks, while dephosphorylation by the phosphatase calcineurin activates Drp1. Active Drp1 constricts and fragments mitochondria. Drp1-driven mitochondrial fragmentation is a critical quality control event upstream of mitophagy

The machinery regulating mitochondrial morphology dynamics is highly integrated with mitophagy initiation. Mitochondrial fission, i.e., division, is mediated by the GTPase dynamin-related protein 1 (Drp1), whereas fusion involves three GTPases; mitofusins 1 and 2 (Mfn1 and Mfn2) mediate outer membrane fusion and optic atrophy 1 (OPA1) mediates inner membrane fusion (Fig. 2) [22]. Upon fission, mitochondria can be segregated into polarized and depolarized daughter mitochondria. While polarized daughter mitochondria can undergo fusion, consistent with the polarization requirement for fusion [23], depolarized mitochondria are targeted by mitophagy [24]. Consistent with this observation, inhibiting the fission machinery or enhancing mitochondrial fusion were both shown to decrease mitophagy [24], while enhancing fission promotes mitophagy [25]. Furthermore, during nutrient starvation-induced autophagy, protein kinase A (PKA)-mediated phosphorylation of Drp1 inactivates fission events, driving the mitochondrial network to an increased fusion state, as a mitophagy inhibition mechanism [26, 27].

Notably, a recent in vivo study has revealed a higher level of complexity. In a conditional Drp1 deletion model in mouse heart, Drp1 knockout increased mitophagy, and promoted dilated cardiomyopathy associated with increased levels of necrotic cell death [28]. Conversely, conditional Mfn1/2 knockout resulted in accumulated dysfunctional mitochondria, lack of mitophagy, and hypertrophy without inducing cell death. These findings not only confirm that mitochondrial dynamics are an essential component of mitophagy, but also expand our understanding past cell culture-based experiments, demonstrating that in vivo the loss of mitochondrial quality control via mitochondrial morphology dynamics is able to engage compensating mitophagy pathways.

## Autophagy induction, maturation, and degradation

**Fig. 3:**
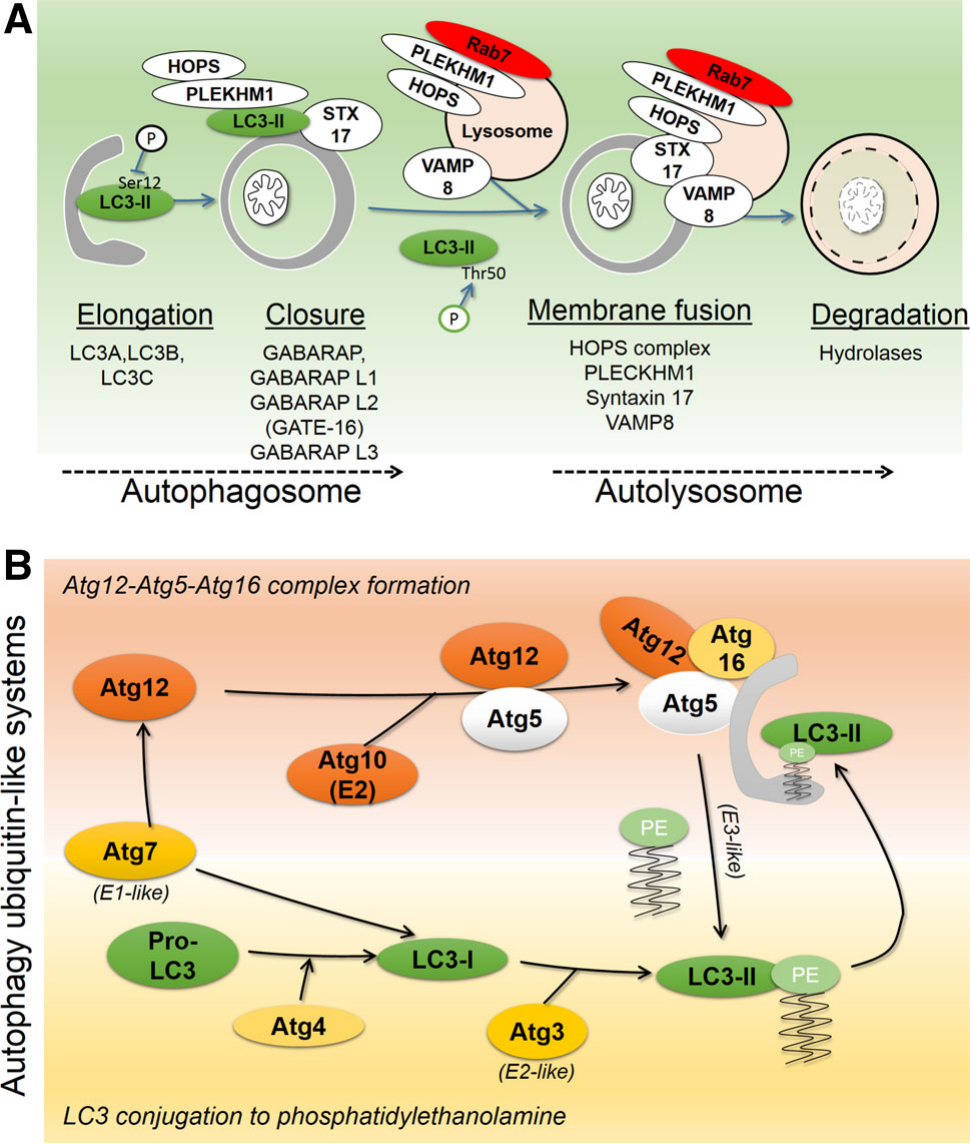
Autophagosome formation and degradation. **a** Autophagy involves phagophore-nucleated autophagosome formation, fusion with endolysosomes to form the autolysosome, and subsequent degradation of the autophagosome and its contents by lysosomal hydrolases. Mammalian Atg8 proteins include proteins of the LC3 subfamily, which participate in phagophore elongation, and of the GABARAP subfamily, which coordinate closure of autophagosome. Proper LC3 function is positively and negatively regulated through phosphorylation. Fusion between autophagosome and endolysosomes is coordinated by Pleckhm1, which at the endolysosome binds Rab7 and the HOPS complex, and at the autophagosome binds LC3 and the HOPS complex. The SNARE member Syntaxin17 (STX17) binds autophagosomes through interaction with the endolysosomal SNARE VAMP8, thereby mediating autophagosomal-lysosomal membrane fusion. Autophagosomes and content are then degraded by lysosomal hydrolases. **b** Cytosolic microtubule-associated protein light chain 3 (LC3)-I is conjugated to phosphatidylethanolamine (PE) to form lipidated LC3-II (LC3-PE), an integral membrane component of the autophagosome and binding partner for autophagy receptors. Two ubiquitin-like conjugation reactions coordinate LC3 lipidation. (1) E1-like and E2-like Atg7 and Atg10 conjugate Atg12 to Atg5, which then complex with Atg16. (2) Pro-LC3 is cleaved at the C-terminus by Atg4, forming LC3-I, which is activated by E1-like Atg7. Through the E2-like Atg3 and E3-like Atg12-Atg5-Atg16 complex, LC3 is lipidated with PE

Macroautophagy (in this review referred to as autophagy) is a catabolic process that in the presence of growth factors and amino acids are negatively regulated by anabolic mTOR signaling. As such, autophagy is repressed under nutrient-rich conditions, and activated under conditions of decreased ATP and nutrients [29]. Upon its induction, the autophagic machinery coordinates the formation and expansion of the so-called phagophore, which encloses cellular proteins and organelles within a double-membraned organelle, the autophagosome. These membrane events are controlled by autophagy-related (Atg) proteins [30]. Autophagosomal membranes likely originate from the endoplasmic reticulum, and may include membrane contents originating from mitochondria, plasma membrane, Golgi, and endosomes [31]. Once formed, the autophagosome fuses with endolysosomes to mature into the autolysosome, whereupon lysosomal hydrolases degrade the autophagosomal inner membrane and its contents [32]. The fusion of autophagosomes with endolysosomes is a highly regulated process (Fig. 3a), and dependent on the GTPase Rab7, which regulates endolysosomal trafficking [33, 34]. Recent findings have shown that maturation is regulated by adaptor proteins which bind autophagosomes and endolysosomes to promote fusion and maturation [35, 36]. The HOPS (homotypic fusion and vacuole protein sorting) tethering complex is responsible for autolysosome formation [37]. HOPS complexes with PLEKHM1 (pleckstrin homology domain containing protein family member 1), which binds Rab7, and can interact with autophagosomes to promote fusion between endolysosomes and autophagosomes [36]. Subsequent membrane fusion events are carried out by SNARE (soluble *N*-ethylmaleimide-sensitive factor attachment protein receptor) proteins; The SNARE Syntaxin 17 binds with the HOPS complex [37], and its interaction with the endolysosomal SNARE VAMP8 mediates fusion of autophagosomes with endolysosomes [38].

## Post-translational modification of Atg8 family proteins during autophagosome formation

A key process in the formation of the phagophore is the conjugation of Atg8 (autophagy-related protein 8) family protein members to phosphatidylethanolamine (PE) [39, 40], an integral membrane component of the autophagosome. In mammals, Atg8 proteins can be divided into LC3 (microtubule-associated protein light chain 3) and GABARAP (gamma-aminobutyric acid receptor-associated protein) subfamilies. The LC3 family includes LC3A, LC3B, LC3B2, and LC3C, and the GABARAP family includes GABARAP, GABARAP-L1, GABARAP-L2/GATE-16, and GABARAP-L3. In the following text, mammalian Atg8 family proteins in general are referred to as LC3. All LC3 family members are integrated into autophagosomes via a C-terminal glycine, covalently conjugated to PE. LC3 conjugation involves two ubiquitin-like reactions (Fig. 3b). The Atg4 cysteine protease first cleaves pro-LC3 at the C-terminus to expose a glycine residue, forming LC3-I. Upon autophagy induction E1 (Atg7) and E2 (Atg3) system conjugates PE to LC3-I, forming LC3-II. Atg12 is conjugated to Atg5 by Atg7 (E1-like) and Atg10 (E2). Atg7 and Atg10 conjugate Atg5 and Atg12, which then forms a complex with Atg16. The Atg5/12/16 complex acts as an E3 ligase, promoting PE conjugation to LC3 [41].

**Fig. 4:**
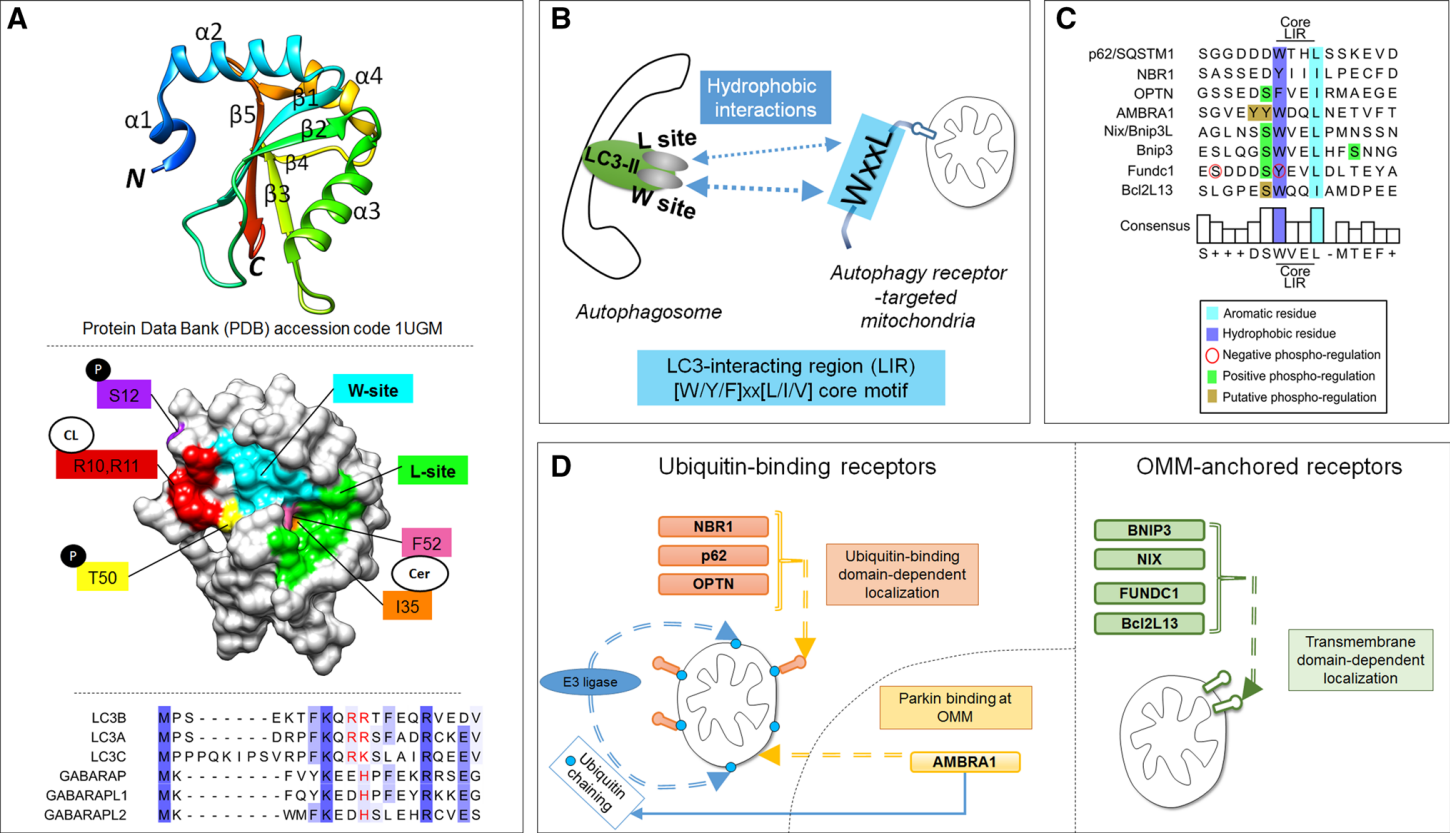
The LC3-interacting region (LIR) motif as a mechanistic basis for mitophagy. **a** LC3B structure (PDB 1UGM) [179], analyzed using Chimera [180]. *Upper panel* ribbon structure diagram indicating α-helices and β-sheets. LC3 proteins contain two N-terminal α-helices and a ubiquitin-like core formed from β strands. Hydrophobic pockets between β2 and α2 form the W-site, and between β2 and α3 form the L-site. *Middle panel* surface representation indicates topology of W- and L-sites (adapted from [49]), phosphorylated residues at S12 and T50, and putative binding sites for cardiolipin (R10 and R11) and ceramide (I35 and F52). *Lower panel*, N-terminal sequence alignments of LC3 homologues. Tcoffee alignment [181] of human LC3 member N-terminal regions using Jalview [182]. *Blue shading* indicates percentage identity, positively charged R, K and H residues, aligning to LC3B R10 and R11, are indicated in *red*. **b** The LIR motif, also known as Atg8 family-interacting motif (AIM), is a short linear peptide motif found in autophagy receptors, which binds LC3-II and thereby underlies selective autophagy. The LIR has a [W/Y/F]xx[L/I/V] core motif, and receptor-ligand interaction occurs through formation of an intermolecular β-sheet via hydrophobic interactions between the LIR motif and the conserved W and L sites on Atg8 proteins. **c** Sequence alignment of reported mitophagy receptor core LIR motifs and neighboring upstream and downstream regions. Phosphorylation can positively or negatively regulate LIR activity. **d** Two main groups of autophagy receptors target mitochondria. E3 ligase-mediated ubiquitylation of OMM proteins recruits ubiquitin-binding, LIR motif-containing receptors, which leads to the binding of receptors and engagement of sequestration by autophagosomes. Alternatively, a group of autophagy receptors contain a transmembrane domain and are constitutively targeted to the OMM. LIR activity of these mitophagy receptors is regulated by phosphorylation events

Upstream, the participation of LC3 proteins in autophagy is regulated by phosphorylation. For instance, integration of LC3B into autophagosomal membranes is suppressed by phosphorylation at serine 12 by protein kinase A (PKA) [42], and STK3/STK4-mediated phosphorylation of LC3B at threonine 50 is needed for efficient autolysosome formation [43] (Figs. 3a, 4a).

While the functional diversity of LC3 proteins has not been entirely established, it has been proposed that LC3 subfamily proteins participate in autophagosomal membrane elongation, while GABARAP subfamily proteins mediate autophagosome maturation [44]. Importantly, LC3 and GABARAP proteins can both serve as binding partners for autophagy receptors, thereby underlying specific modes of autophagy, and permitting autophagy control of diverse cell signaling events, including the antioxidant response [45], pathogen response [46], as well as mitophagy [47].

## LC3 interacting region (LIR) motifs as a basis for specific autophagy

LC3 proteins contain a conserved hydrophobic region, comprised the so-called W and L pockets [48, 49], which docks via hydrophobic interactions with a motif, termed LC3-interacting region (LIR), contained within autophagy receptors [47, 48, 50, 51] (Fig. 4a, b). This LIR motif, also referred to as AIM (Atg8-family-interacting motif) or LC3 recognition sequence (LRS), comprises a core consensus sequence of an aromatic residue followed by a hydrophobic residue [W/F/Y]xx[L/I/V] [47]. This sequence is preceded by negatively charged residues, which are critical for the interaction with positively charged residues on LC3 proteins. Moreover, serine/threonine residues within this LIR preceding region were shown to be fundamental for the regulation of autophagy receptor activity through phosphorylation [46, 52, 53]. So far, it has not been determined if LC3 phosphorylation similarly alters its capacity to bind with autophagy receptors.

During mitophagy, OMM-localized autophagy receptors (mitophagy receptors) attach autophagosomes to the OMM via their LIR motif. To date, eight mechanistically distinct mitophagy receptors have been characterized (Fig. 4c), and can be grouped according to the manner in which they target mitochondria (Fig. 4d). One group of mitophagy receptors contains a ubiquitin-binding domain which localizes them to Parkin-ubiquitylated mitochondria, including p62/SQSTM1, NBR1, and optineurin [54–57]. In addition, mitochondrial-localized Parkin binds AMBRA1 to localize it at the OMM [58, 59]. The second group of mitophagy receptors is made up of Bnip3 [52, 60, 61], its homologue Bnip3L/Nix [62–64], FUNDC1 [53], and Bcl2L13 [65]. These mitophagy receptors contain transmembrane domains and upon expression constitutively localize to the OMM [53, 66–68].

Below we discuss protein mitophagy receptor systems which have been mechanistically elucidated, namely the PINK1/Parkin system, and transmembrane-containing mitophagy receptors. We further discuss the lipids ceramide and cardiolipin, which when localized to the OMM can directly bind LC3 and engage mitophagy [69, 70].

## Mitophagy receptor systems

### The PINK1/Parkin program targets mitophagy receptors to depolarized mitochondria via ubiquitylation of the OMM

**Fig. 5:**
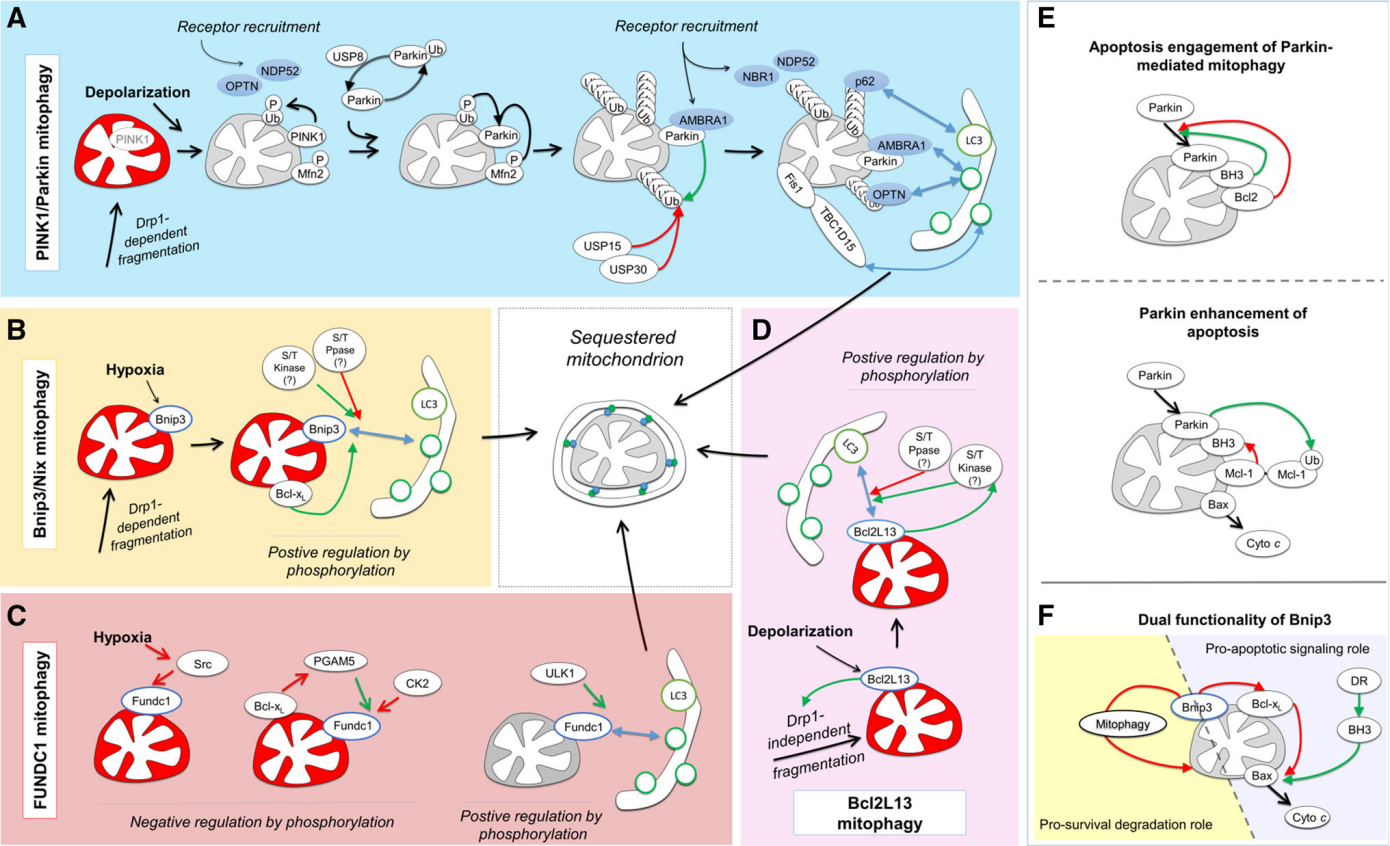
Mitophagy program pathways: triggers and post-translational regulation. **a** Upon mitochondrial depolarization PINK1 is stabilized at the OMM, resulting in phosphorylation of ubiquitin and Mfn2 and consequent recruitment of the E3 ligase Parkin. In the cytosol, Parkin de-ubiquitylation by USP8 is required for mitochondrial translocation. At the mitochondria, Parkin ubiquitylates OMM proteins, resulting in recruitment of ubiquitin-binding autophagy receptors such as p62, OPTN, and NBR1 which then can attach to autophagosomes via their LIR motifs. In addition, PINK1 directly recruits autophagy receptors OPTN and NDP52 through generation of phospho-ubiquitin. AMBRA1 can increase Parkin-mediated mitophagy by direct binding with LC3 and local autophagy stimulation. Sequestration is coordinated by the Rab7 GTPase-activating protein TBC1D15, which localizes to mitochondria by binding Fis1 and binds forming autophagosomes via a LIR. **b** Bnip3 is induced by hypoxia, and localizes to the OMM via one transmembrane domain. Bnip3 LIR activity requires serine 17 phosphorylation, and is enhanced by serine 24 phosphorylation. Responsible kinase(s) and phosphatase(s) have not yet been identified. Pro-survival Bcl-x_L_ activity functions to enhance Bnip3 binding to LC3. Nix contains an identical SWxxL LIR motif and is expected to undergo similar regulation by phosphorylation. **c** FUNDC1 contains three transmembrane domains which accomplish its OMM localization. Under normal conditions, Src kinase phosphorylates tyrosine 18 and thereby inactivates the FUNDC1 LIR. Hypoxia inactives Src kinase, permitting FUNDC1 LIR-mediated binding to autophagosomes. As a second negative regulatory mechanism, CK2 phosphorylates FUNDC1 at serine 13, inhibiting its LIR activity, and PGAM5 phosphatase antagonizes this reaction. The pro-survival Bcl-x_L_ can bind PGAM5, inhibiting its pro-mitophagy activity. Under hypoxia and in response to depolarization serine 17 phosphorylation by ULK1 enhances LIR activity. **d** Bcl2L13 induces Drp1-independent mitochondrial fragmentation and is a LIR-containing mitophagy receptor and mammalian homologue of yeast Atg32 mitophagy protein. **e**, **f** Mitophagy receptor systems undergo crosstalk with mitochondrial apoptosis. **e** Pro-survival Bcl-2 signaling suppresses Parkin translocation to mitochondria, and pro-apoptotic BH3-only proteins can activate Parkin-mediated mitophagy. At longer timescales, Parkin ubiquitylates pro-survival Mcl-1, resulting in its degradation and consequent activation of mitochondrial apoptosis. **f** Bnip3 LIR and BH3 domains confer dual functionality during apoptosis. Under conditions of LIR inactivation, the BH3-only protein Bnip3 increases TNF-mediated caspase activation. Under conditions of engaged LIR activity, the increased degradation of mitochondria can suppress the mitochondrial participation in apoptosis activation. *Green arrows* indicate positive regulation; *red arrows* indicate negative regulation

To date, the best understood mitophagy system is controlled by the serine/threonine kinase PINK1 (PTEN-induced putative kinase 1) and the E3 ligase Parkin1 [71] (Fig. 5a). Herein, PINK1 serves as the sensor for the mitochondrial polarization state. In respiring, polarized mitochondria, PINK1 is imported into the mitochondrial intermembrane space and rapidly degraded through combined activities of the protease PARL (presenilin-associated rhomboid-like protein) and the proteasome [72, 73], thereby maintaining low basal PINK1 levels under normal conditions. Mitochondrial depolarization inactivates its import and proteasomal degradation, leading to PINK1 accumulation on the OMM and resulting in recruitment of Parkin from the cytosol. Parkin translocation to mitochondria has been reported to involve two mechanisms. PINK1 at the OMM phosphorylates Mfn2 at serine 442 and threonine 111, and phosphorylated Mfn2 can act as a receptor to recruit Parkin [74]. In addition, PINK1 phosphorylates ubiquitin at serine 65 [75, 76], and the ubiquitin-like domain of Parkin at serine 65 [77], which drive Parkin recruitment to the OMM and activation of its E3 ligase activity [78]. Once activated and recruited, Parkin E3 ligase activity results in the ubiquitylation of numerous OMM proteins [57, 71], which leads to the recruitment of different LIR-containing autophagy receptors which bind ubiquitin-tagged OMM proteins, including p62/SQSTM1 [79], optineurin [55] and NBR1 [56, 80] (Fig. 4d). Mechanistically, p62 has a role in clustering mitochondria during mitophagy [55, 81, 82], and has been reported to be required [79], or dispensable in downstream mitochondrial degradation [55, 81]. Recently, optineurin was shown to act as the LIR-dependent autophagy receptor downstream of Parkin activation [55]. Furthermore, optineurin can localize TBK1 to p62, and phosphorylate p62 at serine 403 phosphorylation which positively regulates p62 ubiquitin binding [83], generating a feedforward mechanism enhancing p62 targeting of mitochondria. Further, mitochondrial Parkin binds the autophagy-promoting protein AMBRA1 [84]. In response to mitochondrial depolarization, AMBRA1 interacts with Parkin at the OMM and contributes to Parkin-mediated mitophagy via local stimulation of autophagosome formation [58, 59]. Moreover, AMBRA1 was recently shown to bind LC3 through a LIR motif during Parkin-mediated mitophagy, and forced targeting of AMBRA1 to the OMM resulted in efficient depletion of mitochondria, independent of Parkin [85], identifying AMBRA1 as a mitophagy receptor. In addition, in the absence of Parkin, mitochondrial-targeted AMBRA1 was reported to activate mitochondrial ubiquitylation, without recruiting p62. However, the E3 ligase identity, and whether ubiquitylation recruits other ubiquitin-binding autophagy receptors, remains to be determined.

A recent study using TALEN and CRISPR/Cas9 genome editing for combinatorial knockout of five autophagy receptors further consolidates our understanding of the relative contributions of specific autophagy receptors to PINK1/Parkin-mediated mitophagy [86]. Here, p62 and NBR1 were found to be dispensable, and primary but redundant autophagy receptor functions were defined for OPTN and NDP52. Furthermore, PINK1-mediated generation of Ser65 phospho-ubiquitin [75, 76] was revealed as a primary mitophagy signal, autonomously capable of recruiting OPTN and NDP52, thereby proposing a new model within which Parkin functions to amplify PINK1-initiated mitophagy signaling.

#### Deubiquitylation and modulation of Parkin-mediated mitophagy

Ubiquitylation is a reversible process, and recent work has evidenced a central role for deubiquitylating (DUB) enzymes in the regulation of mitophagy (Fig. 5a). Parkin can ubiquitylate itself, resulting in its reduced recruitment to depolarized mitochondria. USP8 deubiquitylation of auto-ubiquitylated Parkin is required for its localization to depolarized mitochondria, and thereby for efficient activation of mitophagy [87]. In addition, acting as negative regulators, the ubiquitin-specific proteases USP30, which localizes at the OMM via a transmembrane domain [88], and USP15, which can fractionally localize to mitochondria, remove Parkin-ligated ubiquitin from OMM proteins [88, 89]. Importantly, both USP30 and USP15 knockdown increased the cellular capacity for mitophagy, suggesting the potential for pharmacological enhancement of mitophagy.

### Constitutively OMM-localized mitophagy receptors are phospho-regulated

In contrast to the PINK1/Parkin mitophagy system, which involves Parkin translocation to mitochondria and subsequent ubiquitin-dependent recruitment of mitophagy receptors, a group of LIR-containing mitophagy receptors, upon expression, constitutively localize at the OMM via transmembrane domains (Fig. 4d). This group of mitophagy receptors is transcriptionally regulated, and engagement of mitophagy receptor activity is controlled through the phosphorylation status of their LIR.

#### Bnip3 and Bnip3L/Nix: OMM-localized mediators of mitophagy and apoptosis signaling

Bnip3 (BCL2/adenovirus E1B 19 kDa interacting protein 3) and its homologue Bnip3L/Nix are atypical members of the pro-apoptotic Bcl-2 subfamily of BH3-only proteins [90], sharing 56 % of the amino acids sequence identity [91]. Both, upon expression, are inserted into the OMM via their C-terminal transmembrane domains, with the N-terminus oriented toward the cytoplasm [66, 92]. At the mitochondria, the BH3 domains of Bnip3 [92] and Nix [93] suppress the function of anti-apoptotic Bcl-2 proteins. In addition to its apoptotic functions, Bnip3 was reported to trigger autophagy [94] and mitophagy [60], and Nix was found to mediate mitochondrial clearance during blood cell development [62, 63]. It was later discovered that both Bnip3 and Nix contain identical N-terminal LIRs (WxxL) [52, 61, 64] (Fig. 4c), thereby providing mechanistic explanation for their mitophagy-inducing function (Fig. 5b).

In addition to this function in coupling OMMs with autophagosomal membranes via their LIR, Bnip3 and Nix undergo crosstalk with the autophagy regulation machinery. Hypoxia-induced autophagy was shown to be supported by Bnip3 and Nix binding to Bcl-2 and consequent disruption of Bcl-2 binding to Beclin 1 [95]. Furthermore, Bnip3 and Nix can bind to the mTOR-activating protein Rheb via their N-terminus, which was associated with reduced activation of mTOR and enhanced autophagy [96]. Under glutamine-mediated enhancement of mitochondrial respiratory activity, Rheb interacted with Nix to induce mitophagy, presumably to meet an increased mitochondrial quality control demand [97]. In this study, Rheb localization to mitochondria did not alter mTOR activity, and it remains to be determined whether Rheb further functions to regulate Nix interaction with LC3.

We have recently shown that the mitophagic activity of Bnip3 is controlled through the phosphorylation state of serine residues adjacent to the LIR [52]. Phosphorylation of serine residues 17 and 24 flanking the Bnip3 LIR specifically promotes binding to LC3B and GATE-16 (Fig. 4c). Interestingly, phosphorylation of Bnip3 at serine 17 is a prerequisite for LC3B and GATE-16 binding, whereas phosphorylation at serine 24 further increased the affinity for both LC3B and GATE-16. Similar to Bnip3, Nix contains a SWxxL LIR motif and activity of its LIR is serine phosphorylation regulated (own unpublished results). To date it remains undetermined which kinases and phosphatases are responsible for controlling the phosphorylation state of the Bnip3 and Nix LIRs.

#### FUNDC1

The OMM-localized protein FUNDC1 contains three transmembrane domains and an N-terminal cytosolic LIR motif (YxxL) for binding to LC3 and GABARAP proteins [53] (Fig. 4c). Also FUNDC1 undergoes positive and negative regulation by phosphorylation, at residues within and preceding the LIR. Under normal conditions, FUNDC1 mitophagy receptor activity is kept in check through inhibitory phosphorylation by Src kinase at tyrosine 18 [53] and by casein kinase 2 (CK2) at serine 13 [98] (Fig. 5c). In response to hypoxia or mitochondrial uncoupling, PGAM5 dephosphorylates CK2-phosphorylated serine 13 of FUNDC1 to activate LC3 binding [98]. In addition, ULK1 phosphorylates serine 17 of the FUNDC1 LIR motif, resulting in increased LC3 binding [99]. Interestingly, Bcl-x_L_ antagonizes PGAM5-mediated dephosphorylation of FUNDC1 and thereby prevents LC3 binding [100], suggesting that in the FUNDC1 system, anti-apoptotic signaling antagonizes the mitophagy response.

#### Bcl2L13/Bcl-Rambo

Bcl2L13 (Bcl2-like 13 or Bcl-Rambo) is an atypical Bcl-2 family member, which contains four BH motifs, but does not bind pro-death or pro-survival Bcl-2 members, and signals apoptosis via its C-terminal transmembrane domain, which targets the OMM [68]. Recently, Bcl2L13 was identified as a mitophagy receptor based on similarity to the yeast mitophagy receptor Atg32, with a WxxL LIR motif [65] (Fig. 4c). Bcl2L13 expression is sufficient to induce mitochondrial fragmentation and target fragmented mitochondria to autophagosomes and endolysosomes (Fig. 5d). All BH domains are required for fragmentation, but in contrast to other modes of mitophagy, fragmentation occurred independent of Drp1. Further, the authors report that CCCP-induced mitochondrial clearance by Bcl2L13 was independent of Parkin, and Bcl2L13 does not induce mitochondrial ubiquitylation in response to CCCP. It is suggested that increased Bcl2L13 levels activate its LIR via phosphorylation: CCCP rapidly increased Bcl2L13 levels, and the mutation of phosphorylation target S272 [101] to alanine reduced total serine phosphorylation of Bcl2L13. Further, while the Bcl2L13 S272A mutant fragmented mitochondria, mitochondria did not co-localize with autophagosomes. Mutating S272 to phospho-mimicking glutamic or aspartic acid residues, and identifying the kinase and phosphatase responsible, will be instrumental for further elucidating the role of Bcl2L13 LIR phospho-regulation.

It is remarkable that all known transmembrane-domain OMM-localized mitophagy receptors contain conserved serine/threonines preceding the LIR (Fig. 4c), indicating that LIR phospho-regulation mechanisms represent a crucial target for altering mitophagy receptor function. Also optineurin contains a serine preceding the LIR, which is required for LC3 binding and its participation in xenophagy [46], rendering it likely that its role in mitophagy is also positively regulated by phosphorylation. Likewise, AMBRA1 contains two tyrosine residues preceding the LIR, which would also be expected to be phospho-regulated (Fig. 4c). Of note, opposed to FUNDC1, we observed that the phosphorylated state of serine 13 of Bnip3 had no impact on LIR activity [52], suggesting that the specific residues preceding the core LIR motif contribute to its regulation.

### Lipid-mediated mitophagy

In addition to protein mitophagy receptors, the lipids ceramide and cardiolipin, when localized to the OMM can directly bind LC3 and engage mitophagy [69, 70]. Increased levels of C18-ceramide at the OMM, either through exogenous addition, or endogenously-generated by ceramide synthase 1 (CerS1), specifically bind to LC3, to induce Drp1-dependent mitophagy [69]. Intriguingly, knockdown of LC3B permitted tumor growth under conditions of increased ceramide production, indicating ceramide-mediated mitophagy promotes cell death. Furthermore, the anti-cancer agent sodium selenite was shown to activate this program via upregulation of CerS1. This intriguing example for a pro-death mode of mitophagy is independent of mitochondrial apoptotic signaling and caspase signaling, and presents a model to elucidate the molecular basis of mitophagic cell death.

Alternatively, cardiolipin, a negatively charged phospholipid, can bind LC3B in response to sub-apoptotic mitochondrial dysfunction. Under normal conditions, most cardiolipin is localized to the IMM, and in response to mitochondrial stress phospholipid scramblase-3 (PLS3) redistributes cardiolipin to the OMM, where it then can bind to LC3B [70]. Interestingly, mutational analysis of LC3B indicates that ceramide interacts with I35 and F52 of LC3B, while cardiolipin undergoes electrostatic interactions with positively charged R11 and R10, which are found in LC3A and LC3B and conserved in the homologous region of LC3C (R16 and K17), but not in members of the GABARAP subfamily (Fig. 4a). Notably, I35 and F52 bind to the LIR of p62, and R10 and R11 bind aspartic acid residues preceding the p62 LIR [51]. However, it remains to be determined whether either of these lipids functionally impact protein autophagy receptor interactions with LC3B.

### Crosstalk between mitophagy receptor systems: a lack of information concerning the level of complexity

It is notable that among the different mitophagy receptor systems, our understanding of crosstalk between mitophagy modes is, so far, restricted to Nix and Parkin pathways. In cell lines, Nix has been shown to promote Parkin translocation to mitochondria, and Parkin-mediated mitophagy [102, 103], and Parkin ubiquitylation of Nix recruits the mitophagy receptor NBR1 [103]. In future work, we propose that mutationally inactivating and activating the LIR using non-phosphorylatable and phospho-mimicking mutations, in combination with genome editing approaches, will serve as fundamental approaches for elucidating whether/which mitophagy receptors form competitive, additive or exclusive programs. Moreover, similar to Parkin, cardiolipin-mediated mitophagy is activated in response to mitochondrial poisons in neuronal cells [70]. Thus, it will be interesting to determine the contributions of lipids to receptor-mediated mitophagy.

## Autophagosomal sequestration of mitochondria

While autophagy receptors capture autophagosomes at mitochondria via their LIR, an outstanding fundamental question for all mitophagy programs is how autophagosomes are localized to dysfunctional mitochondria, and how the sequestration process is coordinated. Upon depolarization, in Parkin-overexpressing cells, autophagy initiating factors, including ULK1, DFCP1 and WIPI-1, localize to mitochondria independently of LC3 [104], mediated by PINK1-recruited autophagy receptors NDP52 and OPTN [86]. Together, these data suggest that autophagosomes are produced at damaged mitochondria and LC3 processing can be localized for the downstream engulfment of mitochondria into autophagosomes. One candidate for such local regulation is AMBRA1 which was shown to exert pro-autophagic activity at mitochondria [58, 59] and additionally contains a LIR [85] which may locally activate and direct autophagosome capture at depolarized mitochondria. In addition, the cellular energy sensor AMPK (5′ AMP-activated protein kinase), which activates autophagy [105] and mitophagy [20], is spatially localized at mitochondria [106], providing a possible sensing mechanism to signal local production of autophagosomes. We observed that expression of a Bnip3 LIR mutant resulted in increased autophagosome content in regions localized next to mitochondria, albeit without mediating sequestration [52], which is consistent with a mechanism of localized autophagosome generation. Recent work has yielded insight into the sequestration process. Rab7 and the Rab GTPase-activating proteins (Rab-GAPs) TBC1D15 and TBC1D17 coordinate autophagosome sequestration of Parkin-targeted mitochondria [107]. TBC1D15 and TBC1D17 bind the OMM-localized, Drp1-binding protein Fis1 and contain LIRs which coordinate autophagosome binding to mitochondria. It remains to be determined if Fis1-TBC1D15 interaction coordinates OMM-localized FUNDC1/Bnip3/Nix-mediated mitophagic sequestration.

Of note, sequestration and degradation of mitochondria requires increased autophagic activity. Thus, the cellular autophagic capacity may be an important limiting factor. Autophagic capacity is under the control of the transcription factor EB (TFEB), which promotes expression of autophagosomal and lysosomal genes [108]. mTOR inhibits TFEB by phosphorylation, thereby coupling autophagy induction and lysosomal activity [109]. Indeed, increased TFEB signaling was shown to enhance Bnip3-induced mitophagy [110] and TFEB together with additional MiT/TFE family members contributes to Parkin-mediated mitophagy [111], supporting that the cellular autophagic capacity is linked to mitophagic efficiency.

## Transcriptional level regulation of mitophagy receptors

**Fig. 6:**
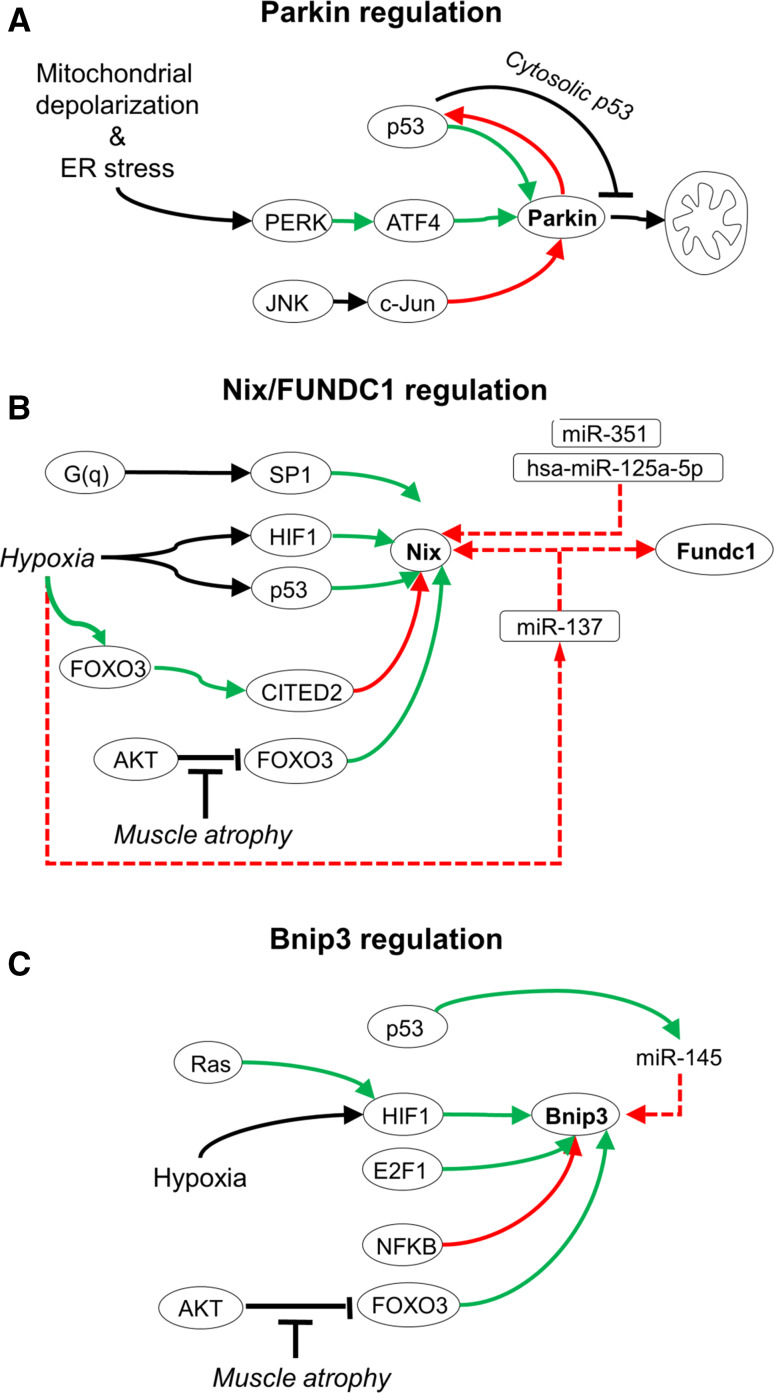
Transcriptional regulation of mitophagy. **a** Under conditions of mitochondrial and endoplasmic reticulum (ER) stress, Parkin expression can be induced by the PERK/ATF4 pathway, and suppressed by the JNK/c-Jun pathway. Further, p53 and Parkin interactions form complex crosstalk. p53 can induce Parkin expression, and Parkin can inhibit p53 expression, forming a negative feedback circuit. In addition, cytosolic p53 can inhibit Parkin translocation to mitochondria. **b** Nix expression is induced by G(q) signaling via SP1, by hypoxia via HIF1 and p53, and FOXO3. Nix expression can be suppressed via FOXO3 activation of CITED2. Both Nix and FUNDC1 are negatively co-regulated by microRNAs. miR-351 and hsa-miR-125a-5p target Nix, and miR-137 co-targets Nix and FUNDC1. **c** Bnip3 expression is increased by Ras activity and induced during hypoxia by HIF1. E2F and FOXO3 activate, and NFkB suppresses Bnip3 expression. *Black arrows* indicate post-translational regulation; *green arrows* indicate transcriptional activation; *red arrows* indicate transcriptional repression

Mitophagy programs are modulated by different transcriptional responses (Fig. 6). Parkin expression is induced by mitochondrial and endoplasmic reticulum stress via the PERK-ATF4 signaling pathway, and dominantly repressed by c-Jun [112]. In addition, nuclear p53 upregulates Parkin expression [113], while cytosolic p53 suppresses Parkin targeting of mitochondria [114], and Parkin suppresses p53 expression [115], suggesting a multi-tiered homeostatic feedback. The mitophagy receptor Bnip3 is prominently regulated at the transcriptional level. Bnip3 expression is driven by hypoxia-inducible factor HIF1 [116–118], which is enhanced by Ras [119] as well as E2F-1 [120], and antagonized by NFκB [121]. Similar to Bnip3, in human tumor cells also Nix is upregulated during hypoxia, via HIF1 [118] and p53 [122]. In cardiac cells on the other hand, Nix is constitutively expressed, and enhanced expression is driven by G_q_-signaling via the transcription factor SP1 [123]. In addition, activated FOXO3 induces Bnip3 and Nix expression in fasting skeletal muscle [124]. However, under hypoxia FOXO3a suppresses HIF1-mediated Nix expression via the transcriptional cofactor CITED2 [125], suggesting negative feedback regulation under hypoxic conditions.

Recently, microRNAs have emerged as negative regulators of the cellular mitophagy capacity. During erythrocyte maturation, the transcriptional repressor KAP1 (Krüppel-associated box (KRAB)-associated protein 1) [125] inhibits expression of miR-351 and hsa-miR-125a-5p, which normally function to suppress Nix and mitophagy [126]. In addition, miR-137 negatively controls expression of Nix and FUNDC1 [127]. Hypoxia-associated down-regulation of miR-137 thereby permits mitophagy receptor expression. In prostate cancer, the p53 inducible miR-145 can suppress Bnip3 expression, thereby countering HIF1 upregulation [128].

## Physiological and pathophysiological implications of mitophagy

As the mechanisms of mitophagy unravel, so do the physiological roles for mitophagy. Several lines of evidence link familiar Parkinson’s disease to impaired mitophagy. Mitochondria are defective in Parkinson’s disease [129, 130]. Deletions and point mutations of Parkin and PINK1 genes (*PARK2* and *PARK6*, respectively) were identified in early-onset Parkinson’s disease patients [71] and these disease-associated mutations were shown to mechanistically result in defective mitophagy [54, 73, 81, 131]. However, extrapolating findings from mechanistic PINK1/Parkin studies to in vivo relevance has been a subject of much debate: Most studies that have elucidated the molecular events of Parkin-mediated mitophagy utilize a combination of artificial Parkin overexpression and chemically-induced mitochondrial depolarization in cancer cell lines. Notably, it was reported that, opposed to in cancer cell lines, mitochondrial depolarization does not lead to robust mitochondrial Parkin translocation in neurons and this was linked to bioenergetics differences between oxidative phosphorylation-dependent neurons and glycolytic cancer cell lines [132]. Moreover, mitochondrial Parkin translocation was shown to occur as a slow and subcellularly restricted process in mature cortical neurons [133], and Parkin accumulation was observed on only a fraction of depolarized mitochondria in neuronal axons [134]. Importantly, it was recently shown that Parkin-mediated mitophagy is constitutively engaged in primary neurons, with PINK1 functioning as the limiting factor for basal mitophagy [89]. In this study, inhibition of USP30, which antagonizes Parkin-mediated mitophagy, increased basal mitophagy by up to fourfold, and in vivo knockdown of USP30 in drosophila was associated with survival. Interestingly, a dominant negative USP30 mutant restored mitophagy in response to PINK1 knockdown, suggesting a fundamental suppressive action. However, it is important to consider that these findings reflect basal neuronal mitophagy activities, and further bioenergetic and oxidative stress-activated studies will be required to determine the extent to which USP30 regulates neuronal mitophagy of dysfunctional mitochondria, and/or autophagy-independent modes of selective mitochondrial degradation [135, 136]. Of note, Parkin knockout mice do not readily exert neurodegeneration or associated motor phenotypes [71], and Parkin knockout in mice with respiratory chain deficiency caused by neuron-specific mitochondrial transcription factor (Tfam) knockout did not impact clearance of mitochondrial aggregates or progression of neurodegeneration [137]. A recent study reports a neuroprotective role for endogenous Parkin, in a model of Parkin knockout mice with a mitochondrial dysfunction background caused by accumulation of mtDNA mutations due to deficiency in DNA polymerase γ [138]. However, while Parkin knockout affected mitochondrial function, no effect on mtDNA mutation burden was detected. Thus, it remains to be determined whether Parkin-mediated mitophagy or alternative functions of Parkin are key to its neuroprotective role in this context.

As mitophagy receptors also Bnip3 and Nix fulfill physiological functions, in addition to their pro-apoptotic function as atypical BH3-only proteins. Bnip3-mediated mitophagy participates in mitochondrial homeostasis in liver of adult mice to avoid metabolic defects [139] and is engaged as a cytoprotective program during ischemia/reperfusion injury in cardiac myocytes [52, 60, 140]. The Bnip3 homologue Nix is transcriptionally upregulated together with Bcl-x_L_ during erythrocyte maturation [141], to drive the removal of mitochondria [62, 63] via its mitophagy receptor function [64]. Furthermore, mouse cytomegalovirus-induced proliferation of antigen-specific natural killer cells is associated with mitochondrial dysfunction, and Bnip3- and Nix-mediated mitophagy were recently shown to be essential in the survival of memory natural killer cells [142]. In heart cells, both PINK1/Parkin- and Bnip3-/Nix-mediated mitophagy were connected to maintaining homeostasis. Parkin-deficiency resulted in enlarged cardiomyocyte mitochondria and respiration defects in *Drosophila* heart tubes [74]. Similar cardiac defects were observed in Mfn2 knockout mice, with a proposed connection to impaired Mfn2-mediated recruitment of Parkin [74]. However, as Mfn2 is a crucial regulator of mitochondrial morphology as well as of mitochondrial-ER junctions, a causative role for altered Mfn2-mediated mitophagy in cardiomyopathy remains to be established. Nix knockout mice developed cardiomyopathy, which developed faster in Bnip3 and Nix double knockout mice [143]. These in vivo findings demonstrate cardioprotective roles for Parkin, Bnip3 and Nix mitophagy programs in the regulation of mitochondrial homeostasis, and further suggest limits to compensating roles. Of note, an important mechanistic distinction from depolarization-driven mitophagy is that Bnip3 can induce mitophagy of polarized mitochondria [52], supporting divergent functional roles between mitophagy programs: In addition to being engaged in order to respond to dysfunctional mitochondria, Bnip3 and Nix appear to be responsible for basal turnover [143], or engaged for developmental [62–64] and immune response programs [142].

### Mitophagy and cancer

Conceptually, a role of mitophagy in cancer is an intriguing hypothesis: Subpopulations of dysfunctional mitochondria can transform cells and promote tumorigenesis [144], suggesting mitophagy could function as a tumor suppressor mechanism. Alternatively, in cancer cells, mitophagy scavenging of pro-apoptotic mitochondria could be cytoprotective. However, to date the roles for mitophagy programs in cancer remain unclear. For example, Parkin may function as a tumor suppressor, as Parkin mutations are common and result in cell cycle deregulation [145, 146]. Furthermore, the expression of Bnip3 and Nix is commonly deregulated in cancer. Both Bnip3 and Nix translation is increased in hypoxic and peri-necrotic tumor regions [118, 147]. High Bnip3 is reported to correlate with invasive tumor behavior in breast [147] and colorectal [148] cancers, and poor prognosis in non-small cell lung [149], prostate [128] and endometrial [150] cancers. In addition, low Bcl2L13 expression correlates with good outcome in childhood acute lymphoblastic leukemia [151]. On the other hand, Bnip3 expression can be silenced in leukaemias [152] and pancreatic [153], colorectal and gastric [154] cancers, and lost Bnip3 [153] or high AMBRA1 [155] expression in pancreatic cancer correlate with worsened prognosis. Thus, these in vivo cancer studies suggest contradicting roles for mitophagy receptors and signaling regulators in cancer, likely due to their simultaneous participation in mitophagy-independent signaling. Therefore, the targeting of specific autophagy receptors in in vivo experiments, for various cancer types and treatment conditions, are required for translational understanding.

Such an approach was recently performed in a mouse model study of metastatic breast cancer [156]. The authors report that deleting Bnip3 promoted tumor growth and malignancy, and resulted in the accumulation of dysfunctional, ROS-producing mitochondria. The resulting increased oxidative stress was shown to activate HIF1α-mediated glycolysis and angiogenesis. Consistently, in human breast cancer low Bnip3 correlated with high HIF1α and poor patient prognosis.

### Role of mitophagy in apoptosis signaling

While it is well established that autophagy and apoptosis signaling undergo pronounced regulatory crosstalk [157], our understanding of the relationship between mitophagy and apoptosis is limited. Recent findings indicate that Parkin undergoes extensive crosstalk with apoptosis pathways (Fig. 5e). Mitochondrial translocation of Parkin was shown to be blocked by pro-survival Bcl-2 proteins, and activated by BH3-only proteins under conditions of inhibited caspase activity [80]. At longer time scales, Parkin can enhance mitochondrial uncoupling-induced apoptosis through degradation of anti-apoptotic Mcl-1 [158]. Consistent with this action, knockdown of USP30, which antagonizes Parkin-mediated mitophagy by deubiquitylating OMM proteins [89], sensitizes cells to activation of the mitochondrial apoptosis pathway [159]. This finding suggests that USP30 would drive mitochondria toward a mitophagy versus apoptosis decision event, and highlights the requirement for further mechanistic understanding.

We further reported that pro-survival Bcl-x_L_ positively regulated the binding of Bnip3 to LC3B, and enhanced mitochondrial sequestration [52] (Fig. 5f). Notably, this regulation differs from FUNDC1, which is negatively regulated by Bcl-x_L_ [100], suggesting separate and mutually exclusive mitophagy pathways.

Functionally, ectopic expression of wild type or LIR-mutated Bnip3 [61], or knockdown of FUNDC1 during hypoxia [53] have no measured impact on apoptosis induction. However, pre-activation of Bnip3-mediated mitophagy through expression of constitutively active Bnip3 receptor prior to TNF (tumor necrosis factor) treatment, significantly reduced effector caspase activation [52]. These findings suggest that enhanced mitophagy activity, and/or delayed activation of mitochondrial outer membrane permeabilization, can reduce the mitochondrial capacity to amplify apoptosis. However, different positive and negative feedbacks between individual mitophagy programs and both pro-survival and pro-death apoptosis signaling, operate at different time scales, and undergo crosstalk [102]. Therefore, it remains to be systematically determined to what extent, in which tissues, and under which (patho)physiological conditions, mitophagy receptors are expressed and regulated. Importantly, the characterized phospho-mimicking mutations [52, 99] will be instrumental in the elucidation of mitophagy receptor function in cell culture and in in vivo models.

## Non-canonical modes of mitochondrial processing by endolysosomes

**Fig. 7:**
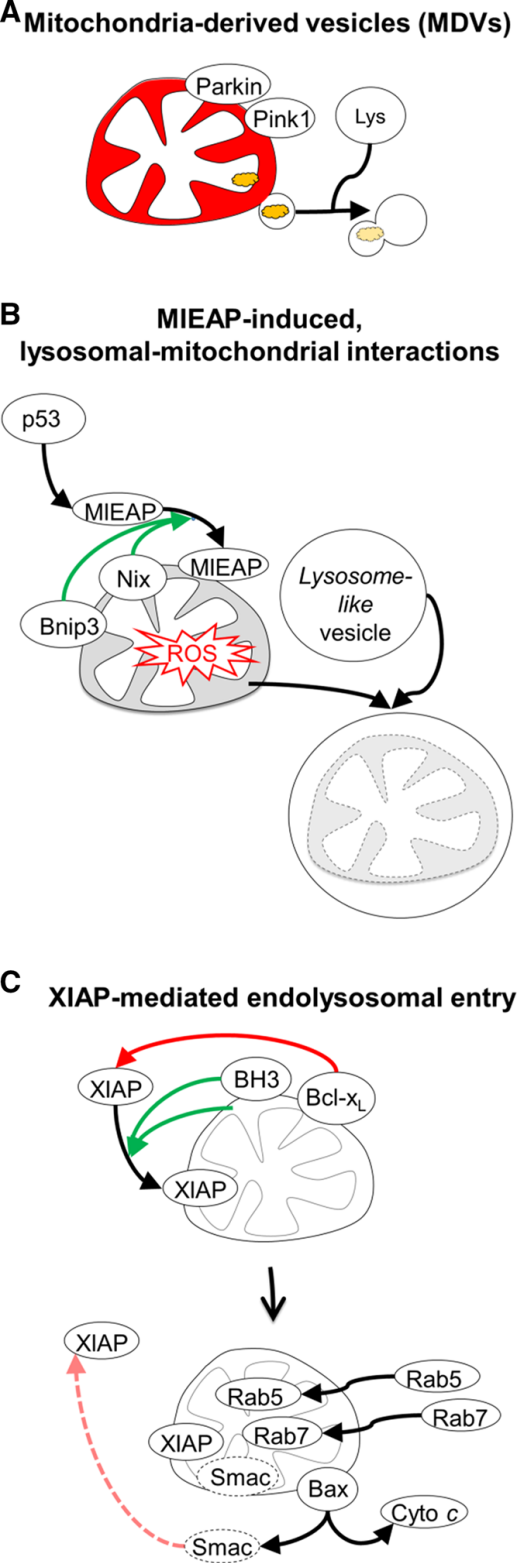
Alternative, autophagy-independent modes of mitochondrial processing by endolysosomes. **a** Under sub-threshold conditions of mitochondrial stress, and prior to depolarization, PINK1 and Parkin mediate a pathway delivering mitochondria-derived vesicles (MDVs), carrying damaged mitochondrial components, directly to endolysosomes. **b** Under conditions of oxidative stress, Nix and Bnip3 can bind with MIEAP (mitochondrial-eating protein), to induce autophagy-independent interactions of mitochondria with lysosome-like organelles. **c** Activity of canonical BH3-only proteins, or mitochondrial depolarization, triggers mitochondrial ubiquitylation by the endogenous caspase inhibitor and E3 ligase XIAP and Bax/Bak-mediated entering of XIAP into mitochondria. Mitochondrial activity of XIAP recruits endolysosomal trafficking machinery into mitochondria, leading to the degradation of its inhibitor Smac, thereby reducing the apoptotic potential of mitochondria

In addition to the above described canonical macroautophagy-mediated modes of mitophagy, mitochondrial stress can engage autophagy-independent endolysosomal interactions with mitochondria (Fig. 7). While chemical-induced mitochondrial depolarization activates PINK1-mediated mitophagy, under conditions of low mitochondrial bioenergetic stress and enhanced oxidative stress, and prior to depolarization, PINK1 and Parkin induce mitochondria-derived vesicles (MDVs) which target oxidized mitochondrial proteins to lysosomes, independently of autophagy [135, 160]. The MDV pathway is proposed to function as a homeostatic quality control mechanism for damage levels which do not require the sacrifice of the entire mitochondrion. In addition, in response to oxidative stress the p53-induced Mieap (Mitochondria-eating protein) induces the autophagy- and Parkin-independent interaction of large endolysosome-like organelles with mitochondria [161, 162]. Mieap activity requires the interaction with Bnip3 and Nix [163], and is proposed to operate in mitochondrial quality control.

We recently reported that expression of the canonical BH3-only proteins tBid, Bim_EL_, Bik and Bad, or treatment with apoptosis inducers TNF or staurosporine, triggers E3 ligase XIAP (X-linked inhibitor of apoptosis protein)-mediated mitochondrial outer membrane permeabilization and XIAP entry into mitochondria [164, 165]. XIAP action at mitochondria results in prominent ubiquitylation at the OMM and IMM, and, intriguingly, autophagy-independent movement of endolysosomal machinery into mitochondria [164]. Inside mitochondria, XIAP catalyzes the proteasome- and lysosome-mediated degradation of its endogenous inhibitor Smac [164], and consequently reduced effector caspase activation during intrinsic apoptosis [165]. Intramitochondrial recruitment of endolysosomes is activated independently of Parkin, Bnip3 or Nix LIR receptor activity. Interestingly, expression of Bnip3 or Nix LIR mutants, with lost mitophagy receptor function, likewise results in activation of this novel pathway [164].

Notably, these three non-canonical modes of mitochondrial processing do not require LC3-decorated autophagosomes, and instead appear to result from direct interorganellar interactions between mitochondria and endolysosomes [135, 161, 164]. One possible advantage over canonical mitophagy, is that direct interactions between endolysosomes and mitochondria can be engaged at faster time scales, as they engage pre-existing organelles.

## Experimental approaches to detecting mitophagy

**Fig. 8:**
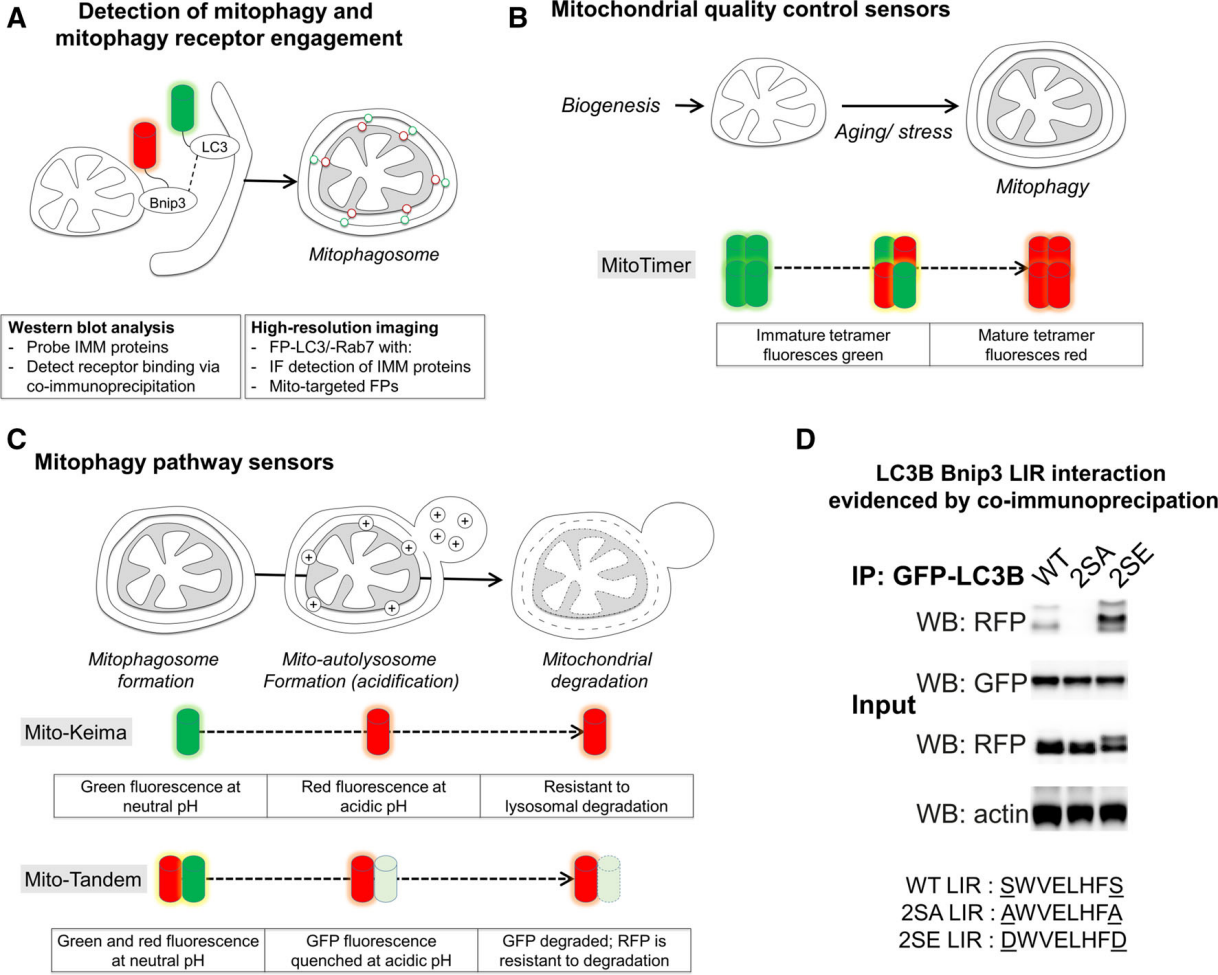
Methodologies to measure mitophagy. Biochemical and imaging-based assays can measure different aspects of mitophagy program activities. **a** Mitochondria-containing autophagosomes can be detected via imaging of fluorescent protein (FP)-LC3-labeled autophagosome colocalization with immunofluorescence or FP-labeled mitochondria. Western blot and immunofluorescence detection of IMM and matrix proteins is most specific for detecting mitophagic degradation events. Mito-autolysosomes can be detected using FP-Rab7. **b** MitoTimer is a tetramer which matures as a green-to-red fluorescent protein. MitoTimer can be used under inducible- and constitutive-expression to analyze mitochondrial quality control dynamics. **c** Mitochondrial entry into the autolysosome and subsequent degradation can be measured using FP sensors targeted to mitochondria, that are sensitive to low pH and resistant to degradation by lysosomal hydrolases. Mito-Keima fluoresces green at neutral pH in the cytosol, and red upon entry into acidic autolysosomes. Mito-Tandem is a mitochondria-targeted RFP–GFP fusion. GFP fluorescence is acid-quenched while RFP fluorescence remains stable also at low pH, permitting *live* cell analysis of mitochondrial presence in autolysosomes. Note, lysosomal hydrolases degrade GFP more efficiently than RFP, permitting detection of mitophagy in fixed cells. **d** Example co-immunoprecipitation demonstrating phospho-regulated Bnip3 mitophagy receptor engagement, i.e., Bnip3 LIR binding with LC3B. MCF7 cells stably expressing GFP-LC3B were transfected with RFP-Bnip3 WT, or LIR-inactive 2SA or LIR-activated 2SE mutants. At 48 h of expression, co-immunoprecipitations (IP) were performed with anti-GFP antibody-coupled magnetic beads. Whole cell lysates (input) and IP samples were analyzed by western blotting (WB). This research was originally published in Zhu et al. [52]. © The American Society for Biochemistry and Molecular Biology

In this section, we highlight assays to detect mitophagy induction and capacity (Fig. 8). Overall, when assessing mitophagy it is important to apply a variety of techniques and to monitor both mitophagy induction and efficiency of degradation.

### High-resolution imaging and biochemical detection and quantification of mitophagy

Mitophagy was first detected using electron microscopy [21], and remains a standard for nanometer analysis of organelle ultrastructure and mitochondrial sequestration. More recently, mitophagy can be directly observed by confocal or widefield fluorescence microscopy, based on sequestration of mitochondria by GFP-LC3-labeled autophagosomes [60] (Fig. 8a). Of note, mitophagy programs vary in the composition of the autophagy machineries. For instance, Nix poorly binds LC3B [64], while Bnip3 preferentially binds LC3B and poorly binds GATE16 [52]. Thus, when assessing novel mitophagy settings, responses of both LC3 and GABARAP subfamilies should be determined. Alternatively, GFP-Rab7 is a suitable tool to detect downstream mitochondrial entry into endolysosomes, independent of LC3 homologue specificity [52] and to capture non-canonical modes of endolysosomal processing of mitochondria [161, 164, 166]. Furthermore, the GFP-LC3-G120A mutant which cannot be conjugated to PE and insert into autophagosomal membranes [39] is applicable to ensure that mitochondrial localization of GFP-LC3 corresponds to autophagosome sequestration [52].

Importantly, when detecting mitochondria using fluorescent dyes, caution and proper controls are paramount. MitoTracker dyes accumulate inside mitochondria based on their membrane potential, and therefore will not efficiently label dysfunctional mitochondria. Moreover, different mitochondrial dyes may be retained differently within mitochondria, dependent on, or independent of, the mitochondrial polarization state [167]. Therefore, strict controls need to be employed to distinguish between changes in mitochondrial mass and the fraction of polarized mitochondria. Alternatively, mitochondria-targeted fluorescent proteins can be used to detect mitochondria [168]. However, also here it needs to be considered that protein import into mitochondria is lost following depolarization [169], possibly resulting in mislocalization of reporter proteins to the cytosol, depending on the experimental time scales. Thus, mitochondria-targeted fluorescent protein sensors may not be suitable for all applications. The use of antibodies targeting endogenous mitochondrial proteins, for mitophagy detection by either immunofluorescence or Western blotting, can circumvent this obstacle. When choosing mitochondrial protein readouts, it is of note that Parkin can activate the proteasomal degradation of OMM proteins in response to CCCP in the absence of mitophagy [170]. Assessing degradation of multiple mitochondrial proteins revealed that cytochrome *c* and VDAC1 are more rapidly degraded, than Complex Va, Complex III Core 1 and cyclophilin D [171], suggesting the latter proteins as more suitable readouts when comparing autophagic capacity between cell types and conditions.

### Mitochondria-targeted maturation-based mitophagy sensors

As a complementary approach, the use of high-content sensors can provide spatio-temporal information concerning mitochondrial biogenesis and lysosomal processing. The MitoTimer sensor (Fig. 8b) was developed based on the tetrameric fluorescent protein drFP583, a derivative of DsRed2, which emits green fluorescence when synthesized, and then progressively shifts to red fluorescence upon oxidation [172]. Two approaches have been developed to extrapolate mitophagy dynamics from MitoTimer fluorescence maturation kinetics. First, using pulsed biosensor expression via a doxycycline-inducible vector, the temporal evolution of red-to-green fluorescence ratio can be used to evidence both mitochondrial turnover and biogenesis [173]. Alternatively, using a constitutive promoter, changes in MitoTimer red-to-green ratio were used to detect changes to mitophagy dynamics in vivo [174]. Of note, while oxidation underlies the green-to-red conversion, it is not fully understood to what extent mitochondrial dysfunction, i.e., ROS production, would influence maturation [172]. A similar tool is mt-Keima (Fig. 8c), which is a mitochondrial matrix-targeted ratiometric pH-sensitive fluorescent protein that fluoresces red at low pH and green at higher pH [175]. Mt-Keima directly reports entry of mitochondria into lysosomes [89], and was used to evidence endogenous activity of Parkin-mediated mitophagy [89].

### RFP–GFP tandem-based mitophagy sensors

An alternative biosensor approach targets a tandem RFP–GFP fusion protein to mitochondria (Fig. 8c), where under *live* cell conditions GFP fluorescence is quenched under acidic conditions of the late endosome/lysosome, while RFP fluorescence remains stable [176]. RFP–GFP targeted to the OMM has been applied to report Parkin-induced mitophagy, and was used for a drug screen, identifying iron chelators as Parkin-independent activators of mitophagy [177]. Similarly, an IMM-targeted RFP–GFP tandem sensor was used to evidence hepatitis B virus-induced activation of mitophagy [178].

For all above fluorescence-based mitophagy assays, an important consideration is that each cell has 100s–1000s of mitochondria, making manual scoring of high-resolution imaging difficult and subjective. Thus, rigorous quantification of single cells and cell populations is required. Moreover, it is necessary to analyze total cellular fluorescence through the *Z*-axis, as the mitophagic response is not uniform throughout all optical slices of a cell. For imaging, the use of high-resolution objectives (60×–100×) and optical sectioning with 300 nm step sizes is advisable. Subsequently, using image analysis software, e.g., Fiji/ImageJ (http://fiji.sc/Fiji), plane-by-plane co-localization can be applied to quantify the extent of mitophagy. Alternatively, images can be manually segmented, and the fraction of mitochondria that is co-localized with autophagosomes can be extrapolated as a fractional cell response. Importantly, a large sample of images must be analyzed to provide meaningful statistics. In addition, high sampling of cells (100s–1000s), as can be achieved using image cytometry [52], or flow cytometry [173], provides a quantitative measure of the number of cells in a population exhibiting a mitophagy response, as well as the degree of response per cell. Of note, cytometry experiments require attention to resolve fluorescence from noise. Modern systems can have up to an 18-bit dynamic range, e.g., a 0–262,144 lower and upper value range. As a rule of thumb, using a log-scale, specific signals should be placed in the four-decade (10^4^/10,000) to five-decade (10^5^/100,000) lower and upper end-linear value ranges to ensure accurate and meaningful measurements.

### Monitoring mitophagy receptor activity using LC3 immunoprecipitation

Finally, a critical tool in the analysis of LIR receptor activities is the immunoprecipitation of tagged-LC3, and analysis of co-precipitated protein by Western blot. Typically this is performed using either of the following two approaches. Cell lysates expressing a receptor of interest can be incubated with purified GST-tagged LC3 proteins [64]. Alternatively, a tagged LC3 (e.g., GFP-LC3), can be co-expressed with a receptor of interest, and immunoprecipitated from the cell lysate [52] (Fig. 8d). Using this latter approach, stable expression of tagged-LC3B/GATE16 in combination with transient transfection of the protein of interest is recommendable to provide best reproducibility. In order to establish LIR-dependency of an interaction with LC3, mutations can be introduced in the putative LIR, e.g., tryptophan (W)-to-alanine (A) and/or leucine (L)-to-A, yielding core LIR-inactive mutants. Serine/threonine(S/T)-to-glutamate (E) or aspartate (D) mutations can be introduced to mimic the phosphorylated state, while S/T-to-A mutation prevents phosphorylation [46, 52]. In addition, basic residues at the W and L hydrophobic pockets of LC3 proteins, e.g., LC3B lysine 49 [49], which interact with acidic/phosphorylated residues preceding the receptor LIR motif, can be mutated to further establish specificity of receptor binding. Of note, mitophagy can be a slow process, and cell line-dependently engaged 24–48 h following transfection.

## Concluding remarks

Mitochondrial dysfunction is implicated in nearly all disease, and recent discoveries suggest paths toward therapeutic manipulation of mitophagy. To that end, the continued elucidation of mechanisms underlying positive and negative mitophagy regulation, as well as roles for mitophagy during normal and stressed conditions will be instrumental in developing treatment strategies. Among the important next steps will be (1) the identification of the kinases and phosphatases that regulate LIR engagement of Bnip3 and Nix to determine in vivo mitophagy significance; (2) the elucidation of how morphology dynamics underlie mitochondrial triage, prior to mitophagy engagement; and (3) an understanding of crosstalk between apoptosis and mitophagy programs, i.e., how mitophagy behavior can regulate mitochondrial participation in programmed cell death. To these ends, RNAi and drug screening using high-content mitophagy sensors will be increasingly important tools in functional and mechanistic elucidation.

## Abbreviations

Atg: Autophagy-related protein
GABARAP: Gamma-aminobutyric acid receptor-associated protein
HIF1: Hypoxia-inducible factor 1
IMM: Inner mitochondrial membrane
LC3: Microtubule-associated protein light chain 3
LIR: LC3-interacting region
mTOR: Mammalian target of rapamycin
OMM: Outer mitochondrial membrane
PE: Phosphatidylethanolamine
ROS: Reactive oxygen species
TCA: Tricarboxylic acid
TFEB: Transcription factor EB
